# Simple and multiple relationships between assertiveness, sensation seeking, Alexithymia and addiction potential in university students – RETRACTION

**DOI:** 10.1192/j.eurpsy.2025.26

**Published:** 2025-02-26

**Authors:** Z. Alayi

This abstract, which was originally published as part of volume 28.S1, Abstracts of the 21^st^ European Congress of Psychiatry, 2013, has been retracted with the agreement of the journal editors due to concerns around potential plagiarism.
